# Structural and histological characterization of oviductal magnum and lectin-binding patterns in G*allus domesticus*

**DOI:** 10.1186/1477-7827-9-62

**Published:** 2011-05-08

**Authors:** Jin Gyoung Jung, Whasun Lim, Tae Sub Park, Jin Nam Kim, Beom Ku Han, Gwonhwa Song, Jae Yong Han

**Affiliations:** 1 Avicore Biotechnology Institute, Optifarm Solution Inc., Gyeonggi-Do 435-050, Korea; 2 WCU Biomodulation Major, Department of Agricultural Biotechnology, Seoul National University, 599 Gwanak-ro, Gwanak-gu, Seoul 151-921, Korea

## Abstract

**Background:**

Although chicken oviduct is a useful model and target tissue for reproductive biology and transgenesis, little is known because of the highly specific hormonal regulation and the lack of fundamental researches, including lectin-binding activities and glycobiology. Because lectin is attached to secreted glycoproteins, we hypothesized that lectin could be bound to secretory egg-white proteins, and played a crucial role in the generation of egg-white protein in the oviduct. Hence, the purpose of this study was to investigate the structural, histological and lectin-binding characteristics of the chicken oviductal magnum from juvenile and adult hens.

**Methods:**

The oviductal magnums from juvenile and adult hens were prepared for ultrastructural analysis, qRT-PCR and immunostaining. Immunohistochemistry of anti-ovalbumin, anti-ESR1 and anti-PGR, and mRNA expression of egg-white genes and steroid hormone receptor genes were evaluated. Lectin histochemical staining was also conducted in juvenile and adult oviductal magnum tissues.

**Results:**

The ultrastructural analysis showed that ciliated cells were rarely developed on luminal surface in juvenile magnum, but not tubular gland cells. In adult magnum, two types of epithelium and three types of tubular gland cells were observed. qRT-PCR analysis showed that egg-white genes were highly expressed in adult oviduct compared with the juvenile. However, mRNA expressions of *ESR1 *and *PGR *were considerably higher in juvenile oviduct than adult (*P *< 0.05). The immunohistochemical analysis showed that anti-ovalbumin antibody was detected in adult oviduct not in juvenile, unlikely anti-ESR1 and anti-PGR antibodies that were stained in both oviducts. In histological analysis, Toluidine blue was stained in juvenile and adult oviductal epithelia, and adult tubular glands located in the outer layer of oviductal magnum. In contrast, PAS was positive only in adult oviductal tubular gland. Lectins were selectively bound to oviductal epithelium, stroma, and tubular gland cells. Particularly, lectin-ConA and WGA were bound to electron-dense secretory granules in tubular gland.

**Conclusions:**

The observation of ultrastructural analysis, mRNA expression, immunohistochemistry and lectin staining showed structural and physiological characterization of juvenile and adult oviductal magnum. Consequently, oviduct study could be helped to *in vitro *culture of chicken oviductal cells, to develop epithelial or tubular gland cell-specific markers, and to understand female reproductive biology and endocrinology.

## Background

The oviduct of oviparous animals such as chicken and quail is an amazing organ. It produces each structural component of the laid egg, including the egg-white and eggshell. The mechanisms underlying the egg-laying process are sensitively regulated by steroid hormones, which orchestrate the proliferation and growth of oviductal epithelial cells. For example, diethylstilbestrol (DES) administration leads to massive growth of the juvenile oviduct [1] and induces cytodifferentiation of epithelial cells into tubular gland cells, goblet cells, and ciliated cells [2]. The oviductal magnum is regarded as an important target tissue [3,4] for transgenic research and the production of glycosylated pharmaceutical proteins in chickens because most egg-white proteins are synthesized and secreted in the magnum segment to the oviductal lumen during the 24-h egg production cycle, and this process is mediated by a series of hormones.

The egg-laying hen oviduct is divided into several parts: the infundibulum (place of fertilization), magnum (place of egg-white protein production), isthmus (formation of the shell membrane), shell gland (formation of the egg shell), and vagina (oviposition). While the oviduct mucosa of 10-week-old juvenile chickens is simply lined by a thin layer of pseudostratified columnar epithelial cells upon compact stroma cells [2], the oviductal magnum mucosa from egg-laying hens consists of surface oviductal epithelium lined by ciliated non-secretory cells, non-ciliated secretory granular cells (also referred to as goblet cells), and three different types of tubular gland cells under the epithelium [5]. The ciliated cells rarely show secretory activity and consist of cilia in the luminal mucosa [6] but non-ciliated cells are mainly involved in the release of secretory granules that are synthesized by tubular gland cells.

On the other hand, granular cells have a unique intracellular structure of highly conserved glycoprotein and actively release the egg-white protein mass into the lumen when an egg is proceeding through the magnum segment [6]. Glycoprotein, carbohydrates, and lectin have been commonly shown to have different distributions and binding patterns depending on the species, age, sexual maturity, and hormonal effects [7-12]. In addition, these materials are involved in sperm binding to the oviductal epithelium [13,14], sperm trapping in the oviductal mucosa [15,16], and secretory activity of oviductal ampulla during the estrous cycle [10]. Despite the importance of lectin and carbohydrates in reproductive biology, little is known about chicken oviduct. Traditionally, the characterization of the chicken oviduct has been limited to immunohistochemical staining against egg-white proteins, including ovalbumin, ovomucoid, lysozyme [17] and steroid hormone receptors [18,19].

Although chicken is regarded as a useful tool for transgenesis as a bioreactor [3,20-22], the production mechanism of recombinant humanized proteins are not well understood because of difficulties of transgenic chicken production, lack of *in vitro *verification system of transgene and fundamental researches of chicken oviduct, and highly sensitive hormone reaction in oviduct. In this study, we conducted a series of experiments using electron microscopy, quantitative RT-PCR, immunohistochemical analysis, and lectin histochemistry in juvenile oviductal magnum and that of egg-laying hens. The results obtained in this study should aid in our understanding of bird reproduction, mechanism of egg-white protein production, glycosylation, and the *in vitro* culture of chicken oviductal cells.

## Methods

### Experimental animals and animal care

The care and experimental use of chickens was approved by the Institute of Laboratory Animal Resources, Seoul National University (SNU-070823-5). Chickens were maintained according to a standard management program at the University Animal Farm, Seoul National University, Korea. The procedures for animal management, reproduction, and embryo manipulation adhered to the standard operating protocols of our laboratory.

### Scanning electron microscopy (SEM) and transmission electron microscopy (TEM)

The magnum segment of chicken oviducts from juvenile (10-week-old) and actively egg-laying (30-week-old) hens were obtained, fixed primarily at 4°C for 2-4 h with modified Karnovsky's fixative (2% glutaraldehyde and 2% formaldehyde in 0.05 M sodium cacodylate buffer, pH 7.2), washed three times with cacodylate buffer, fixed secondarily for 2 h with 1% osmium tetroxide in cacodylate buffer, and stained overnight with 0.5% uranyl acetate at 4°C. To observe specimens for scanning electron microscopy (SEM), samples were dried twice with 100% isoamyl acetate for 15 min in a critical point dryer, mounted on metal stubs, coated with gold, and observed under field emission (FE)-SEM (SUPRA 55VP; Carl Zeiss). To prepare specimens for transmission electron microscopy (TEM), samples were dehydrated through a graded ethanol series, embedded in Spurr's resin, and cut on an ultramicrotome (MT-X; RMC, Tucson, AZ, USA). Samples were then stained with 2% uranyl acetate and Reynold's lead citrate for 7 min each and observed under TEM (LIBRA 120; Carl Zeiss).

### Total RNA extraction and real-time PCR analysis

Total RNA was extracted from the oviduct and muscle samples from juvenile (10-week-old) and egg-laying adult (30-week-old) chickens using TRIzol according to the manufacturer's instructions (Invitrogen, Carlsbad, CA, USA). Extracted RNA was quantified using a spectrophotometer and 1 ug of each RNA sample was reverse-transcribed into 20 μl of single-stranded cDNA using the Superscript III First-Strand Synthesis System (Invitrogen). Primer sets were synthesized to amplify specific fragments of chicken oviductal transcripts as described in Table 1. To analyze the expression patterns of oviduct-specific genes, the iCycler iQ Real-Time PCR Detection System (Bio-Rad, Hercules, CA, USA) and EvaGreen (Biotium, Hayward, CA, USA) were used for quantitative RT-PCR. Non-template wells without cDNA were included as negative controls and each test sample was run in triplicate. The PCR amplification was performed at 94°C for 3 min, followed by 35 cycles at 94°C for 30 s, 60°C for 30 s, and 72°C for 30 s, using a melting curve program (increase in temperature from 55°C to 95°C at a rate of 0.5°C per 10 s) and continuous fluorescence measurement. Relative quantification of gene expression was calculated after normalization of the transcript to *GAPDH *(endogenous control) and the nonspecific control using the 2^-ΔΔCt ^method. The PCR products were also loaded on a 1% agarose gel with ethidium bromide.

**Table 1:** Primer sequences for RT-PCR

Gene Name	Sequence (5' to 3'): Forward and Reverse	**GenBank Accession No**.	Product Size (bp)
ovalbumin	tgagcatgttggtgctgttg ttttcctccatcttcatgcg	NM_205152.1	154
ovomucoid	agcgaggacggaaaagtgat cctgctctactttgtgggca	NM_001112662.1	118
lysozyme	gctctggggaaagtctttgg gcggctgttgatctgtagga	NM_205281.1	192
avidin	caggcacctacatcacagcc tcaggacctccttcccattc	NM_205320.1	192
estrogen receptor alpha	gtccatctgctggaatgtgc aagatttccaccatgccctc	NM_205183.1	149
progesterone receptor	cagccagagctcccagtaca gacagcagttcctcaagcga	NM_205262.1	249
gapdh	acgccatcactatcttccag cagccttcactaccctcttg	NM_204305.1	578

### Immunohistochemistry and lectin staining

The oviductal magnum segments of juvenile (10-week-old) and egg-laying adult (30-week-old) chickens were fixed in 4% buffered paraformaldehyde after strong washing with phosphate-buffered saline (PBS). Segments were subsequently embedded into a paraffin block and the paraffin-embedded oviductal tissue was sectioned at a thickness of 6 μm. The deparaffinized and rehydrated samples were heated in a microwave for 10 min after immersion in a sodium citrate buffer solution at pH 6.0 for heat-induced epitope retrieval (HIER). For immunohistochemical analysis, samples were permeabilized with 0.1% Triton X-100 in PBS for 5 min and incubated with 0.1% normal goat serum for 1 h to block nonspecific binding. Samples were serially stained at 4°C for overnight by indirect labeling using the following primary antibodies; mouse anti-chicken OVA antibody (1:200 dilution; Sigma), rabbit anti-human ESR1 antibody (1:100 dilution; Sigma), and mouse anti-human PGR antibody (1:100 dilution; Biocare Medical, Concord, CA, USA). To detect the primary antibodies, an AP detection system (Dako Universal LSAB2 kit; DakoCytomation, Carpinteria, CA, USA) was conducted [23] and then samples were observed under an inverted microscope (TE2000-U; Nikon).

For lectin histochemistry, the oviductal samples were reacted with FITC-conjugated lectins (Sigma) such as peanut agglutinin (PNA), *Helix pomatia *agglutinin (HPA), concanavalin A (ConA), *Ulex europaeus *agglutinin-1 (UEA-1), *Wisteria floribunda *agglutinin (WFA), and wheat germ agglutinin (WGA) at 10 μg/ml [9,24] for 30 min. For double staining, juvenile and adult oviductal samples were incubated with anti-OVA, anti-PGR and anti-ESR1 antibodies at 4°C for overnight, respectively, and serially reacted with 10 μg/ml Cyanine (Cy) 3 or PE-conjugated anti-mouse IgG or rabbit IgG antibodies for 1 h at RT, and each samples were incubated with FITC-conjugated lectin-WGA for 30 min. These fluorescent samples were counterstained with diamidino-2-phenylindole (DAPI) and observed under a confocal microscope (LSM-700; Carl Zeiss, Wetzlar, Germany).

### Histochemistry

For histological and chemical dye staining, sections were stained with hematoxylin-eosin (HE; Sigma, St. Louis, MO, USA) for 2 min and 20 min, respectively [23], Periodic acid-Schiff's staining (PAS; Sigma) for 5 min and 15 min, respectively, or Toluidine blue (TB; Sigma) for 2 min. All procedures were performed at room temperature, and stained cells were observed under an inverted microscope (TE2000-U; Nikon, Tokyo, Japan).

### Statistical analysis

The PROC-GLM model of the SAS program (SAS Institute, Cary, NC, USA), which employs an analysis of variance (ANOVA) and the least-squares method, was used to statistically analyze the numerical data, i.e., oviductal samples from juveniles and adults, and muscle samples as controls. A significant difference was determined when the *P *value was less than 0.05.

## Results

### Ultrastructure of the oviductal magnum from juvenile and egg-laying adult chickens

**Figure 1:**
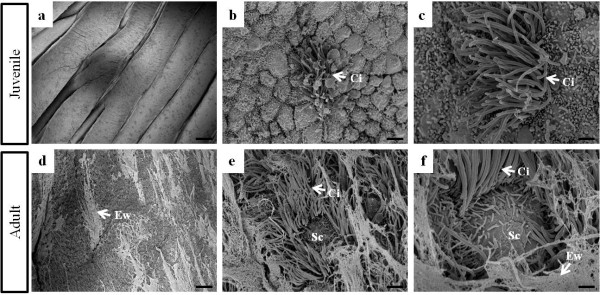
**Scanning electron microscopy (SEM) of oviductal magnum from juvenile (10-week-old) and egg-laying adult (30-week-old) chickens**. The juvenile oviductal magnum was composed of an epithelia bundle (a) covered with a few ciliated non-secretory cells (b, c). In the adult oviductal magnum (d-f), the luminal surface was covered with well-developed cilia and bulging non-ciliated secretory cells, including a large mass of secreted egg-white materials. Ci, cilia; Ew, egg-white; Sc, secretory cells. Bars = 100 μm (a), 10 μm (d), 2 μm (b, e), 1 μm (c, f).

**Figure 2:**
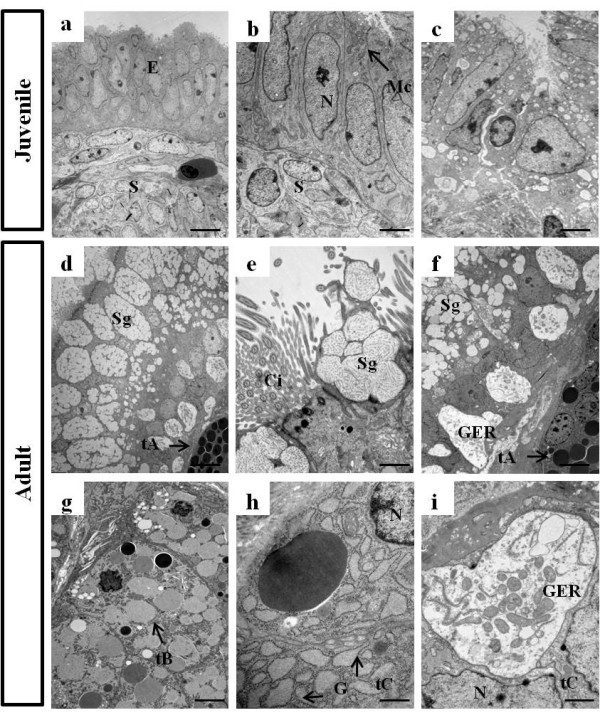
**Transmission electron microscopy (TEM) of oviductal magnum from juvenile (10-week-old) and egg-laying adult (30-week-old) chickens**. The oviductal magnum from the juvenile was simply composed of an epithelial layer toward the luminal surface and stroma cells underneath the epithelium (a-c). In the epithelium, numerous mitochondria (Mc) were observed (b). The adult oviductal magnum was composed of epithelia on the luminal surface and tubular gland cells (d-i). The secretory epithelia contained large masses of homogenously processed secretory granules (d, e). Tubular gland cells generally contained electron-dense granules in the tubular gland. Type A (tA) cells had homogenous electron-dense granules (d, f), type B (tB) cells contained low electron-dense granules (g), and type C (tC) cells possessed well developed GER cisternae and a Golgi complex to transform electron-dense granules to secretory granules (h, i). Ci, cilia; E, epithelium; G, Golgi complex; GER, granular endoplasmic reticulum; Mc, mitochondria; N, nucleus; S, stroma; Sg, secretory granule. Bars = 10 μm (a), 5 μm (b,c), 2 μm (d), 1 μm (f, g), and 100 nm (e, h, i).

To examine the ultrastructural properties and differences in both juvenile and adult oviductal magnum, we performed field emission-scanning electron microscopy (FE-SEM) and transmission electron microscopy (TEM) analyses. As shown in Figure 1a, FE-SEM showed that the oviductal magnum from juvenile chickens was formed by a flexible luminal bundle, separated by deep furrows. In addition, non-secretory ciliated cells were rarely developed in the luminal bundle of the surface lamina propria of the juvenile oviductal magnum (Figure 1b, c). However, the luminal epithelium of the egg-laying hen oviductal magnum was filled with well developed ciliated columnar secretory and non-secretory ciliated cells, bulging secretory granular cells, and a secretory egg-white protein mass, which were prominent and bulged on the luminal surface of the magnum when observed under the scanning electron microscope (Figure 1d-f). The study of ultrastructure of oviducts indicated that the magnum epithelia of the juvenile chicken oviduct were not cytodifferentiated into tubular gland cells and adult oviductal magnum was certainly filled with ciliated secretory cells and secreted egg-white protein mass in the lumen. Furthermore, as illustrated in Figure 2, TEM revealed that the surface of the glandular lumen from the juvenile oviduct was simply composed of an epithelial layer toward the luminal surface and stroma cells underneath the epithelium (Figure 2a-c). However, the surface of the adult oviductal lumen was lined with columnar epithelial cells (also known as goblet cells; Figure 2d) and tubular gland layers (Figure 2f-i). The surface epithelium were composed of non-secretory ciliated epithelial cells and secretory non-ciliated granular cells (Figures 1e, f and 2e). The surface epithelial cells secreted egg-white proteins to the glandular lumen. Secretory granules were also observed in the surface of the granular openings at the time of egg-white protein mass secretion (Figure 1d and 2e) and the surface of the oviductal lumen was mostly covered with the secretory egg-white protein mass (Figure 1d, f). Our current data demonstrated that the tubular gland cells were divided into three different types as previously reported [25]. As shown in Figure 2d-i, type A cells were filled with electron-dense granules, type B cells contained a large mass of homogenous amorphous materials of low electron density, and type C cells were occupied by granular endoplasmic reticulum (GER) cisternae with a large and prominent Golgi complex around the nucleus, which those cells obviously participate production of egg-white proteins in tubular gland. This comparative ultrastructural study of the juvenile and adult oviductal magnum will allow us to better understand the chicken oviductal epithelia and tubular gland, which synthesize and secrete egg-white proteins

### Magnum-specific gene expression in both juvenile and egg-laying adult chickens

**Figure 3:**
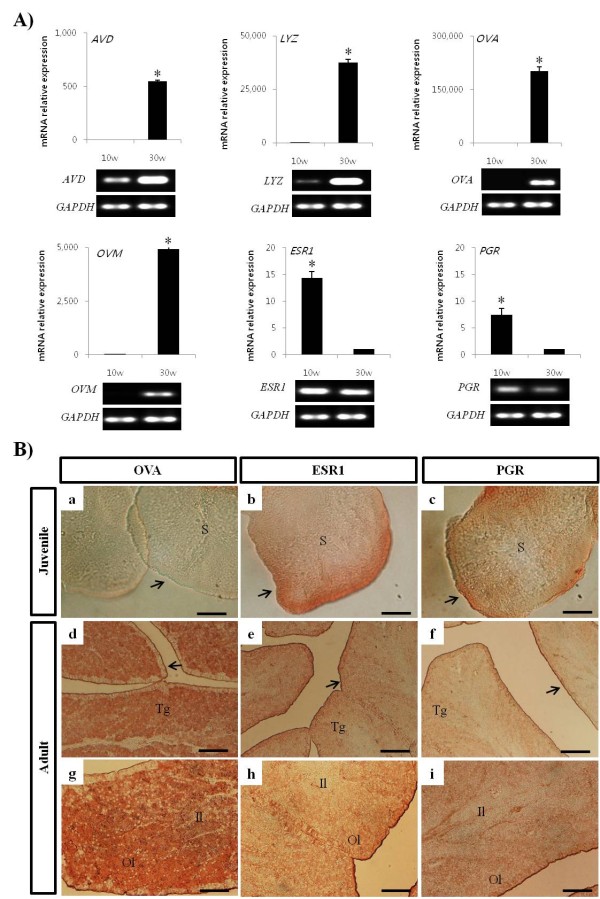
**Oviduct-specific gene expression and immunohistochemical analysis from juvenile (10-week-old) and egg-laying adult (30-week-old) oviducts**. A) The competitive RT-PCR of egg-white-specific genes including *OVA, AVD, OVM*, and *LYZ*, and hormone receptor-derived genes including *ESR1 *and *PGR *were analyzed. PCR products were also loaded on agarose gels with ethidium bromide. The competitive RT-PCR analysis (*P < 0.05) showed that the expression levels of oviduct-specific genes in the adult oviduct were significantly higher than those in the juvenile oviduct. However, the relative expression levels of *ESR1 *and *PGR *in the juvenile oviduct were significantly higher than in the adult oviduct (*P < 0.05). B) Oviductal magnum sections from the juvenile and the adult were immunostained with anti-OVA, anti-ESR1, and anti-PGR antibodies. In the juvenile oviduct (a-c), rarely stained anti-OVA result (a) was observed compared with the immunoreactivities of anti-ESR1 (b) and anti-PGR antibody (c), which strongly stained the epithelium (arrows). In the adult oviduct (d-i), anti-OVA antibodies mainly bound to the tubular gland (Tg; d, g). Anti-ESR1 antibody bound to the oviductal epithelium and toward the outer layer (Ol) of tubular gland compared with the inner layer (Il) of tubular gland (e, h). The anti-PGR antibody was positive to the tubular gland and epithelium (f, i). E, luminal surface of the oviductal epithelium; Il, inner layer of the tubular gland; Ol, outer layer of the tubular gland; S, stroma; Tg, tubular gland. Bars = 100 μm (d-f), 50 μm (g-i), and 25 μm (a-c).

**Figure 4:**
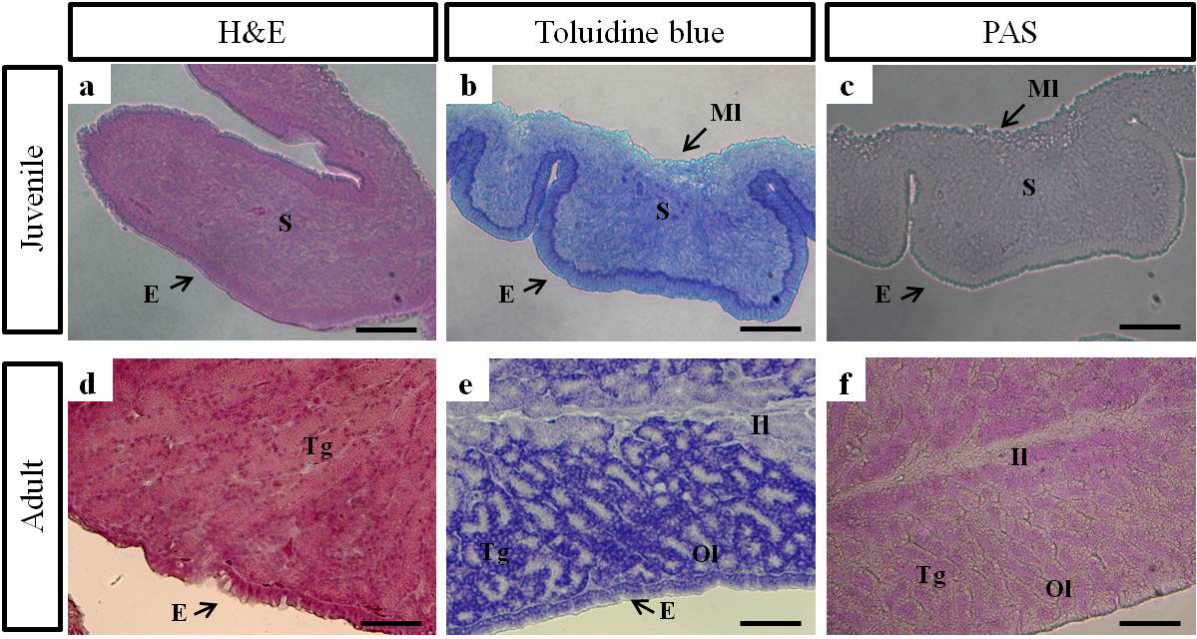
**Histochemical analysis of the oviductal magnum from juvenile (10-week-old) and egg-laying adult (30-week-old) chickens**. Oviductal magnum sections were stained with hematoxylin-eosin (HE; a, d), Toluidine blue (TB; b, e), and Periodic acid Schiff's (PAS) solution (c, f) from juveniles (a-c) and adults (d-f). In the juvenile oviduct, TB strongly stained in the surface epithelium (b) but PAS staining was not observed (c). In the adult oviductal magnum, TB was strongly bound toward the outer layer of the oviductal magnum. This suggests that TB was bound to the processed egg-white that were ready to be secreted into the lumen. PAS was broadly stained to both layers of the oviductal magnum. E, luminal surface of the oviductal epithelium; Il, inner layer of the tubular gland; Ml, muscularis layer; Ol, outer layer of the tubular gland; S, stroma; Tg, tubular gland. Bars = 50 μm.

To evaluate the oviductal magnum-specific gene expression in the oviduct, secretory egg-white genes, including ovalbumin (*OVA*), ovomucoid (*OVM*), lysozyme (*LYZ*), and avidin (*AVD*), and hormone receptor genes, such as estrogen receptor α (*ESR1*) and progesterone receptor (*PGR*), were measured in the oviduct from juvenile and adult chickens. As shown in Figure 3A, the mRNA expression of secretory egg-white protein genes in the adult oviductal magnum was significantly higher than the juvenile oviductal sample except for *ESR1 *and *PGR*. A 549-fold increase of *AVD *(juvenile versus adult, *P *< 0.05), 37,557-fold increase of *LYZ *(*P *< 0.05), 202,155-fold increase of *OVA *(*P *< 0.05), and 4,931-fold increase of *OVM *(*P *< 0.05) were observed, which suggests that egg-white protein genes were barely expressed in juvenile oviduct compared with adult oviduct. However, mRNA expression of *ESR1 *and *PGR *in the juvenile oviduct was considerably higher than the adult oviduct, and showed a 14.3-fold increase of *ESR1 *(adult versus juvenile, *P *< 0.05) and 7.2-fold increase of *PGR *(adult versus juvenile, *P *< 0.05) (Figure 4A). These expression profiles were sequentially confirmed in the oviducts of 4-week-old, 10-week-old, and 15-week-old chickens and the similar expression patterns were obtained (data not shown).

### Immunohistochemical analysis in the oviductal magnum from juvenile and egg-laying adult chickens

To confirm the result of mRNA expression, immunohistochemical analyses were conducted with anti-OVA antibody, the most abundant secretory egg-white protein, and anti-hormone receptors antibodies, such as anti-ESR1 and anti-PGR antibodies. Figure 3B showed that anti-OVA antibody was barely bound to the juvenile oviduct, but abundantly and predominantly localized on the tubular gland layer in the adult oviduct. Notably, anti-ESR1 and anti-PGR antibodies were detected in the apical surface of the luminal epithelium of both the juvenile and adult oviduct and these antibodies were also localized on the tubular gland layer in the adult oviduct (Figure 3B).

### Histological characterization of oviductal magnum from juvenile and egg-laying adult chickens

The paraffin-embedded magnum samples were stained with HE, TB, and PAS reagents (Figure 4). Morphologically, the magnum of the juvenile oviduct was simply lined by surface luminal epithelium, subepithelial stroma, and an outer longitudinal muscularis layer (Figure 4a-c). The juvenile oviduct had not completely developed the fine structures of a typical adult oviduct, which developmentally classified with epithelial cells and tubular gland cells. In the juvenile oviduct, TB was specifically stained the basal layer of the luminal epithelium. However, in the adult oviductal magnum, TB was strongly stained the pyramidal cells of the tubular glands with acidophilic cytoplasm and flat basal nuclei. This result suggests that TB was bound to the processed egg-white materials that were ready to be secreted into the lumen. In addition, PAS did not stain the juvenile oviduct, but predominantly stained both outer and inner layer of the tubular gland from the adult oviductal magnum. In adult, there was a critical difference between PAS reagent for glycogen and proteoglycan, and TB reagent for proteoglycan and glycosaminoglycan. This staining result indicates that secretory granules in the tubular gland layer can be divided into the outer and the inner layers and contain different types of polysaccharides, e.g., glycogen or proteoglycans in the inner layer and proteoglycans or glycosaminoglycan in the outer layer.

### Lectin histochemical stating in the oviductal magnum

**Figure 5:**
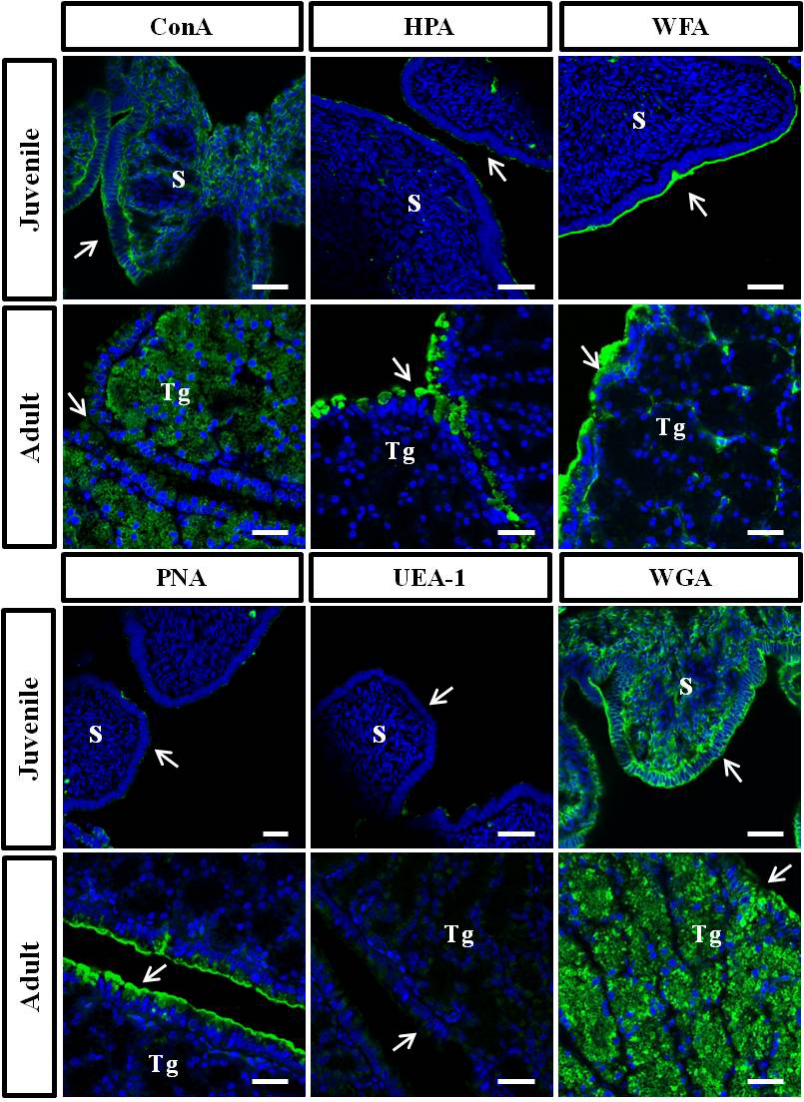
**Lectin-histochemical analysis by laser confocal microscopy in juvenile (10-week-old) and egg-laying adult (30-week-old) hen oviducts**. Oviductal magnum sections were stained with FITC-conjugated ConA, HPA, WFA, PNA, UEA-1, and WGA, and counterstained with DAPI for nuclear staining. Arrows, luminal epithelium; S, stroma; Tg, tubular gland. Bars = 25 μm.

To investigate lectin-binding patterns in the juvenile and egg-laying hen oviducts, FITC-conjugated lectins, including mannosyl (ConA), glucosyl (ConA), *N*-acetylglucosaminyl (WGA), sialic acid (WGA), galactosyl (PNA), *N*-acetylgalactosaminyl (HPA, WFA), and fucosyl (UEA-1) residues (Table 2), were used for histological observations. As shown in Figure 5, lectins ConA and WGA were mainly located in the surface epithelium and stroma cell layer of the juvenile oviduct, whereas lectins HPA, PNA, and UEA-1 were barely detected. Moreover, the lectin WFA residue was abundantly and specifically detected on the apical surface of the luminal epithelium, but not the stroma cells. The egg-laying hen oviduct showed very different lectin-binding patterns as compared with the juvenile oviduct. Lectins ConA and WGA were abundantly and predominantly detected in the tubular gland cells (Figure 5). Interestingly, HPA and PNA residues were predominantly found on the apical surfaces of the luminal epithelium, but rarely identified in the tubular gland. Similarly, the WFA residue was specifically distributed in the apical surface of the luminal epithelium and the boundaries of the gland tubules. However, lectin UEA-1 was barely detected. Consequently, both the juvenile and adult oviductal epithelium were found to contain specific sugar residues, including mannosyl, glucosyl, *N*-acetylgalactosaminyl, galactosyl, *N*-acetylglucosaminyl, and sialic acid (Table 2). In contrast, electron-dense secretory granules in the tubular gland of the adult oviduct presumably expressed mannosyl, glucosyl, *N*-acetylgalactosaminyl, *N*-acetylglucosaminyl, and sialic acid (ConA and WGA).

**Table 2:** Summary of lectin-binding patterns in the juvenile and adult oviductal magnum

Lectin	Source of Lectin	Specific Residues	Juvenile Oviduct		Adult Oviduct	
			Epithelium	Stroma	Epithelium	Tubular Gland
ConA	*Canavalia ensiformis*	Mannosyl, glucosyl	+++	++	+	+++
HPA	*Helix pomatia*	*N*-acetylgalactosaminyl	+/-	+/-	+++	+/-
WFA	*Wisteria floribunda*	*N*-acetylgalactosaminyl	+++	+/-	+++	+
PNA	*Arachis hypogea*	Galactosyl	+/-	+/-	+++	+/-
UEA-1	*Ulex europaeus-1*	Fucosyl	+/-	+/-	+	+
WGA	*Triticum vulgaris*	*N*-acetylglucosaminyl, sialic acid	+++	+++	+++	+++

ConA, concanavalin A; HPA, *Helix pomatia *agglutinin; WFA, *Wisteria floribunda *agglutinin; PNA, peanut agglutinin; UEA-1, *Ulex europaeus *aggulutinin-1; WGA, wheat germ agglutinin

**Figure 6:**
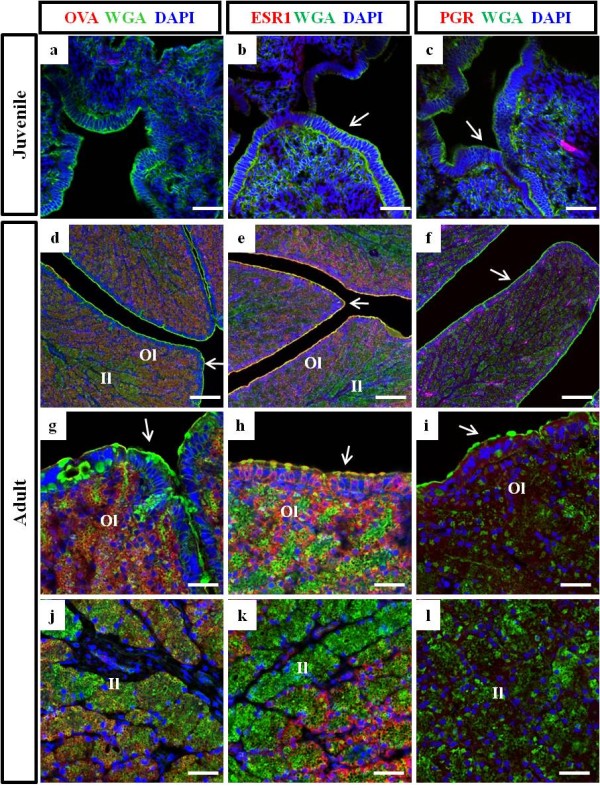
**Immunohistochemical analysis by laser confocal microscopy of the oviductal magnum from juvenile (10-week-old) and egg-laying adult (30-week-old) chickens**. Oviductal magnum sections were immunostained with anti-OVA, anti-ESR1, and anti-PGR, and co-stained with lectin-WGA and DAPI. In the juvenile oviduct, anti-ESR1 and anti-PGR antibodies stained the epithelium slightly and rare staining occurred with the anti-OVA antibody. In the adult oviduct, the anti-OVA antibody stained the tubular gland layers located toward the outer layer (d, j), but not the luminal epithelium, which stained for lectin WGA only (g). The anti-ESR1 antibody strongly stained the tubular gland layers toward the outer layer (e, k) and luminal epithelium (h), but not the tubular gland in the inner layer (k). The anti-PGR antibody broadly stained the tubular gland (f, l) and epithelium (i). Arrows, luminal surface of the oviductal epithelium; Il, inner layer of the tubular gland; Ol, outer layer of the tubular gland. Bars = 100 μm (d-f) and 25 μm (a-c and g-l).

In next experiment, the double-staining was also conducted with antibodies for OVA, ESR1 and PGR, and FITC-conjugated lectin WGA (Figure 6). Lectin WGA was broadly bound to the tubular gland, apical surface, and basal layer of luminal epithelium in the adult oviduct. The anti-OVA antibodies was strongly stained the tubular gland located in the outer layer of the oviductal magnum (Figure 6d), but not in the luminal epithelium which was positive for lectin WGA only (Figure 6g). The anti-ESR1 antibody was strongly stained the tubular gland located in the outer layer and luminal epithelium (Figure 6e, h), but not the inner layer of the tubular gland (Figure 6k). The anti-PGR antibody was stained both the tubular gland (Figure 6f, l) and epithelium (Figure 6i).

## Discussion

In this study, it revealed that the chicken oviductal magnums from juvenile and egg-laying adult hens were physiologically and functionally different, based on the studies of the ultrastructural analysis, quantitative RT-PCR analysis, immunohistochemical analysis of ovalbumin and steroid hormone receptors, and lectin histochemistry. Our results also indicated that the juvenile oviductal magnum was not differentiated into functional tubular gland cells, even though ciliated non-secretory cells were rarely observed on the luminal surface.

In chicken, juvenile oviductal magnum is simply lined up by undifferentiated oviductal epithelia, which could be cytodifferentiated into tubular gland cells by estrogen [1]. Two types of columnar epithelial cells on the surface of the granular lumen and three types of tubular gland cells located under the luminal epithelium of the magnum mucosa were observed in the oviductal magnum from the egg-laying hen. Ciliated non-secretory cells were broadly scattered and covered the surface of the glandular lumen. Non-ciliated secretory cells were surrounded by numerous ciliated cells and secreted large masses of egg-white components such as ovalbumin. In the oviductal magnum, tubular gland cells are classified into another three different types: type A cells, filled with electron-dense granules; type B cells, filled with a large mass of homogenous material with low amounts of electron-dense granules; and type C cells, which are occupied by GER cisternae and a large and prominent Golgi area. The type C cells are regarded as recovered type A cells that have transferred their granules during passage of the egg [26]. In the present study, we confirmed previous studies of the adult oviductal magnum comparing with the ultrastructural observations on the juvenile magnum region.

During development of the chicken oviduct, oviduct-specific gene expression and cytodifferentiation of epithelial to tubular gland cells is mainly triggered by steroid hormones. Basically, estrogen initiates the differentiation of progenitor cells of the epithelium into tubular gland cells in the magnum [1,27,28]. These cells then synthesize and secrete large amounts of major egg-white proteins (ovalbumin, conalbumin, lysozyme, and ovomucoid) [29]. It is therefore no wonder that chicken oviductal epithelial cells express steroid/nuclear hormone receptors, including PGR and ESR [18,19,30,31], which are induced by primary stimulation of estrogen and secondary stimulation of estrogen, progesterone, and glucocorticoids [1,27,32]. In this study, the mRNA expression levels of *ESR1 *and *PGR *in the juvenile oviductal magnum were significantly higher than those of the adult oviductal magnum. This result represents *ESR1 *and *PGR *are expressed in the oviductal epithelia and may receive steroid hormone signals, which regulate the vigorous proliferation and cytodifferentiation from the epithelium to the tubular gland at the juvenile stage. These signals result in a massive increase in oviduct size and weight before sexual maturation. However, directly comparing *ESR1 *and *PGR *mRNA expression in the juvenile and adult oviductal magnum was difficult because cell types and their populations in juvenile and adult oviduct were obviously different. Furthermore, anti-OVA, anti-ESR1, and anti-PGR antibodies were more strongly and obviously bound to the outer layer of the tubular gland, compared with the inner layer of adult oviduct. In contrast, lectin WGA and ConA were localized in the tubular gland cells of both layers of tubular gland. This result suggests that tubular gland cells located in the inner layer are not activated and differentiated enough to secret granules as compared with those in the outer layer of the tubular gland.

With respect to reproductive biology, lectins are known to act as functional molecules that regulate cell adhesion binding to glycoproteins. Lectins allow the sperm reservoir to interact with the oviductal epithelium [33]. Specifically, they bind to a soluble carbohydrate or to a carbohydrate moiety that is part of extracellular and intracellular glycoproteins. In numerous studies on the mammalian oviduct, lectins have been used to detect a variety of carbohydrate residues such as mannose [34], fucose [34], galactose (*N*-acetylgalactosamine) [35-37], *N*-acetylglucosamine [38], and *N*-acetylneuraminic acid (sialic acid) [38]. These studies also revealed that lectins such as Con A (concanavalin A; α-D-mannose and α-D-glucose) [34], HPA (*Helix pomatia *agglutinin; D-*N*-acetyl-galactosamine) [35,36], LTA (*Lotus tetragonolobus *agglutinin; α-L-fucose), RCA 1 (*Ricinus communis *agglutinin 1; β-D-galactose), UEA-1 (*Ulex europaeus *agglutinin-1; α-L-fucose) [39], and WGA (*Triticum vulgaris *agglutinin; D-*N*-acetyl-glucosamine, and sialic acid) [38] can be exploited to identify certain components such as epithelial cell types in the oviduct. Those lectins play a crucial role in the binding of spermatozoa to epithelial cells and gamete interactions [8]. Lectin-binding sites on the oviduct show different patterns depending on age, region, sex cycle, and estrous cycle [14,40,41]. Lectin studies, however, have primarily focused on the mammalian oviduct, and little is known regarding the chicken oviduct and glycoconjugates. In the present study, we revealed that lectins are selectively bound to the oviductal epithelium, stroma, and tubular gland layers. Particularly, lectin WGA and ConA bound to the electron-dense tubular gland cells in the chicken oviduct, which means that secretory granules of tubular gland are contained *N*-acetylglucosaminyl, sialic acid, D-mannosyl, and D-glucosyl residues in adult oviduct. These results also indicate that changes in hormonal responsiveness in the oviductal magnum during development can generate differences in the expression of sugars and glycosylation patterns of egg-white proteins [42]. We hypothesize that these carbohydrate modifications might be involved in oviduct-specific gene expression, such as ovalbumin, and sperm adhesion for the fertilization. However, further studies are necessary to confirm this postulate.

In the biopharmaceutical industry, glycosylation is critically related to protein reactivity and modulates the efficacy of therapeutic proteins [43,44]. The production of human pharmaceutical glycosylated proteins derived from mammalian cell lines have limited production capacity and require glycoengineering processes to add *N*-linked glycosylation [45]. Potential advantages of using transgenic chickens as bioreactors include the simplistic egg mixture, which is composed of approximately 11 major proteins, the massive production of eggs, and similarities with the glycosylation of *N*- and *O*-linked glycans of humans as compared with other mammals [46,47], which leads to a reduced potential risk for adverse immune responses to pharmaceutical proteins produced in eggs [48]. For example, a study examining the glycosylation of IgGs in different species revealed that IgG from cows, sheep, and goats contain oligosaccharides with *N*-glycoyslneuraminic acid (NGNA), whereas humans and chickens only incorporate *N*-acethylneuraminic acid (NANA, referred to as sialic acid) [47], which shows a prolonged serum half-life and increased biological activity [49]. Pathways for both *N*- and *O*-linked glycosylation are highly activated in the tubular gland cells of the oviduct, which secrete egg-white proteins that are almost all glycosylated. In this study, lectin-binding patterns in egg-laying hens demonstrated the need for combination studies examining glycosylation profiles of lectins and egg-white proteins and their precursors in the oviductal magnum. These profiles could provide a better understanding of the glycosylation of pharmaceutical proteins generated from transgenic chickens, including *N*-linked glycan and sialic acid, because the target tissue for producing recombinant proteins is the oviductal magnum. However, further studies would be necessary to characterize egg-white proteins and their precursors in the tubular gland of the oviductal magnum, classify sialic acid and *N*-acetyl-glucosamine that bind to lectin WGA, and identify the glycosylation profiles of therapeutic proteins from the oviduct.

Consequently, it is the first study to combine ultrastructural analysis, immunohistochemistry, and lectin-binding patterns of the juvenile and adult oviductal magnum in chickens. This study contributes to our understanding of the mechanisms underlying avian reproductive biology and transgenesis. In addition, these results can help to conduct further studies such as *in vitro *culture of oviductal cells, development of novel markers, glycoengineering for bioreactors, female reproductive biology, and immortalized cell-lines for producing exogenous proteins *in vitro*.

## Competing interests

The authors declare that they have no competing interests.

## Authors' contributions

JGJ designed and performed all the experiments and drafted the manuscript. WL contributed to the tissue sampling. TSP and GS participated in the design of the study, data analysis and drafted the manuscript. JNK and BKH carried out experimental animal management and participated manuscript writing. JYH, as a corresponding author, designed the experiments, analyzed experimental data and drafted the manuscript. All authors read and approved the final manuscript.
